# SPARC plays an important role in the oviposition and nymphal development in *Nilaparvata lugens* Stål

**DOI:** 10.1186/s12864-022-08903-z

**Published:** 2022-10-03

**Authors:** Weixia Wang, Tingheng Zhu, Pinjun Wan, Qi Wei, Jiachun He, Fengxiang Lai, Qiang Fu

**Affiliations:** 1 State Key Laboratory of Rice Biology, China National Rice Research Institute, Hangzhou, 310006 China; 2 College of Biotechnology and Bioengineering, Zhejiang University of Technology, Hangzhou, 310014 China

**Keywords:** *Nilaparvata lugens*, SPARC, RNAi, Parental RNAi, Fat body

## Abstract

**Background:**

The brown planthopper (*Nilaparvata lugens* Stål)is a notorious rice pest in many areas of Asia. Study on the molecular mechanisms underlying its development and reproduction will provide scientific basis for effective control. SPARC (Secreted Protein, Acidic and Rich in Cysteine) is one of structural component of the extracellular matrix, which influences a diverse array of biological functions. In this study, the gene for *SPARC* was identified and functionally analysed from *N.lugens*.

**Results:**

The result showed that the *NlSPARC* mRNA was highly expressed in fat body, hemolymph and early embryo. The mortality increased significantly when *NlSPARC* was downregulated after RNA interference (RNAi) in 3 ~ 4^th^ instar nymphs. Downregulation of *NlSPARC* in adults significantly reduced the number of eggs and offspring, as well as the transcription level of *NlSPARC* in newly hatched nymphs and survival rate in progeny. The observation with microanatomy on individuals after *NlSPARC* RNAi showed smaller and less abundant fat body than that in control. No obvious morphological abnormalities in the nymphal development and no differences in development of internal reproductive organ were observed when compared with control.

**Conclusion:**

*NlSPARC* is required for oviposition and nymphal development mainly through regulating the tissue of fat body in *N.lugens*. *NlSPARC* could be a new candidate target for controlling the rapid propagation of *N.lugens* population. Our results also demonstrated that the effect of *NlSPARC* RNAi can transfer to the next generation in *N.lugens*.

**Supplementary Information:**

The online version contains supplementary material available at 10.1186/s12864-022-08903-z.

## Introduction

Rice brown planthopper (*Nilaparvata lugens* Stål) (Hemiptera: Delphacidae), a major sap-sucking pest, causes the wilting and complete drying of rice (*Oryza sativa*), a condition known as ‘hopperburn’ under heavy infestations [1]. Strategies for controlling *N.lugens* include breeding resistant cultivars and using synthetic pesticides. However, continued cultivation of resistant varieties has allowed the emergence of new virulent biotypes of *N. lugens* that can overcome existing resistance genes [2, 3]. The use of synthetic pesticides can kill natural predators and ultimately develops insecticide resistant biotypes [4, 5]. Therefore, more economical, targeted, and sustainable control methods are highly needed to pest management. The discovery of important functional genes required for the growth and development of pests through research is an effective strategy to explore potential targets for pest control.

SPARC (Secreted Protein, Acidic and Rich in Cysteine) is one of the structural components of the extracellular matrix, which has been shown to bind calcium and collagens, modulate cell–matrix interactions and influence a diverse array of biological functions. SPARC has been the topic of a number of reviews [6–8]. In vertebrate species it participates in bones mineralization or regulating cell proliferation in some cancer types. *SPARC* mis-regulation perturbs the function of many extracellular matrices and associated tissues, and correlates with cancer progression [9, 10]. *SPARC* has been studied only in a limited number of invertebrate species. In the nematode *Caenorhabditis elegan*s, downregulation of *SPARC* expression using RNA interference (RNAi) demonstrated it affects the function of a variety of tissues and is required for completion of normal development [11]. In the cnidarian *Nematostella vectensis*, four *SPARC* orthologs have been identified and their expression is restricted to the endoderm in early stages of development [12]. Among insects, *SPARC* has been thoroughly studied in the fly *Drosophila melanogaster* with results revealing multiple functions in oogenesis, vitellogenesis, embryo development, heart formation and cardiomyopathies [13–18]. In the cockroach *Blattella germanica*, depletion of *SPARC* does not allow the follicular cells to complete mitosis, resulting in a great alteration of the ovarian follicle cytoskeleton and disability of females for oviposition [19]. However, no studies on *SPARC* in *N.lugens* have been reported to date.

In this study we cloned the *SPARC* gene from *N.lugens*. The contribution of *SPARC* to the development and reproduction in *N.lugens* was investigated by using microanatomy and RNAi technology to knock down its expression in different developmental stages, including early and late instar nymphs, newly emerged adults and gravid females.

## Results

### *NlSPARC* identification

Based on *N. lugens* transcriptome data constructed in our laboratory and genome data from National Center for Biotechnology Information (assembly ASM1435652v1), one specific PCR primer pairs for *SPARC* were designed and used to clone this gene from *N.lugens*. The cDNA of *N.lugens SPARC* (*NlSPARC*) consists of 1288 nucleotides, including the 5′ and 3′ untranslated regions, and an open reading frame of 891 nucleotides (from 138 bp to1028 bp). It ensodes a protein of 296 amino acids, with a theoretical molecular weight of 34.7 kDa and an isoelectric point of 5.3. The cDNA sequences of *NlSPARC* were deposited in GenBank under accession number MZ983402. Blast against NCBI showed that *NlSPARC* shared high identity with the SPARC predicted from *Thrips palmi* (up to 70.9% identity with an E value 4e-74 and 45% coverage). Blast analysis with *N.lugens* genome data showed that *NlSPARC* was located on chromosome 2 and was composed of at least 7 exons.

**Fig. 1:**
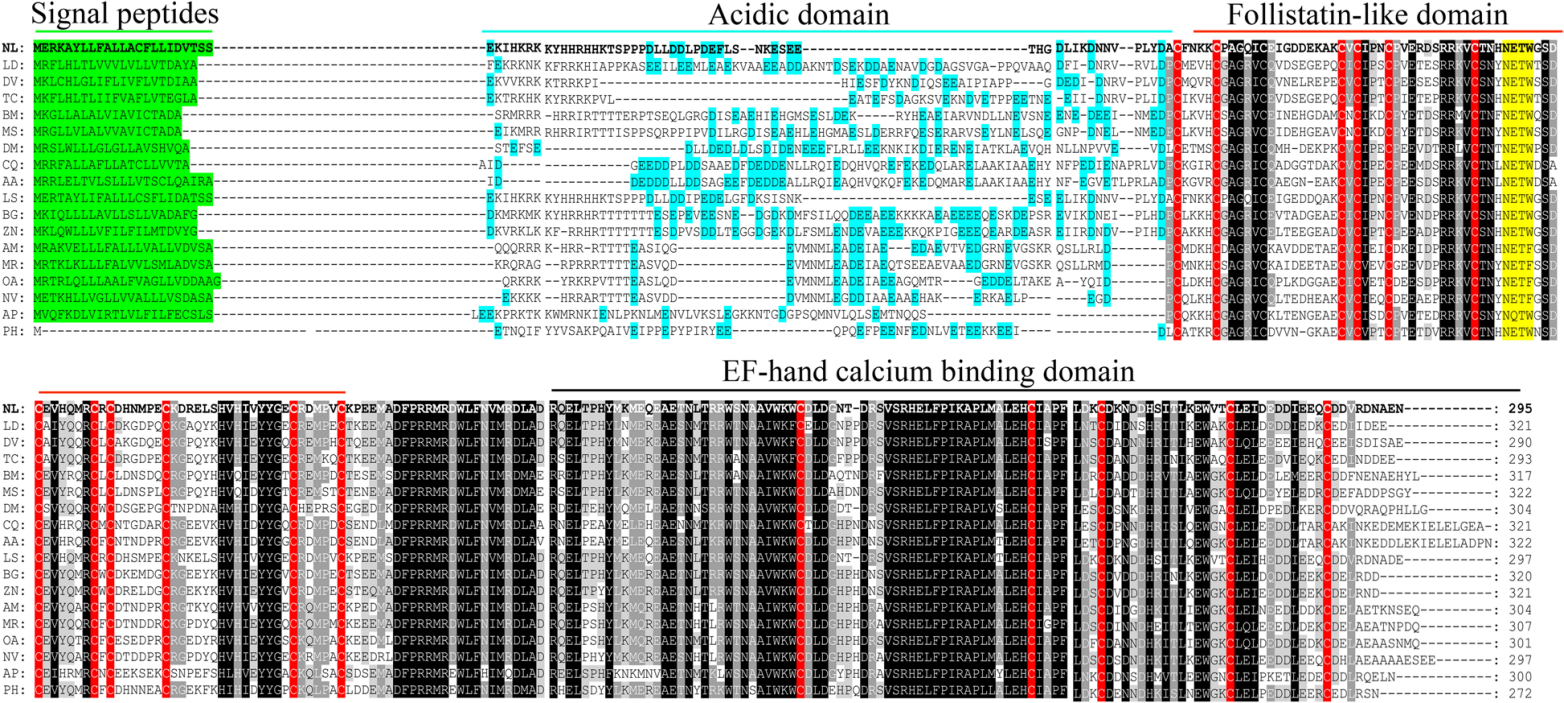
Amino acid sequences alignment of NlSPARC. The amino acid position is indicated on the right. Identical residues between orthologous sequences are shown as white characters against black background, and conservative substitutions are shaded. In green is indicated the signal peptides. In yellow is indicated the conserved glycosylation site. In red are highlighted the cysteine residues. In blue is indicated the acid residues. The *N.lugens* SPARC sequence is highlighted with bold letters. All protein sequences obtained from GenBank are listed in Table S1

The sequence of the NlSPARC protein is similar with other insect SPARC orthologs, showing 87.5% and 70.6% identity to the SPARC from *Laodelphax striatellus* and *Blattella germanica*, respectively. When compared with SPARC from *D. melanogaster*, its identity decreases to 50.0%. As in other insect SPARC proteins, NlSPARC is structured in the three characteristic domains, Domain I (the acidic domain in the N-terminal), Domain II (the follistatin-like domain, the conserved putative N-glycosylation site) and Domain III (EF-hand calcium binding domain). NlSPARC sequences begin at the N-terminal methionine of the signal sequences (MERKAYLLFALLACFLLIDVTSS) (Fig. 1). Domain I (residues 24–76) have 12 acidic residues, the overall acidity is reduced by the presence of 9 basic residues (pI 6.3). NlSPARC contains 16 cysteine residues, 11 of which are conserved in domain II (residues 77–191). The C-terminal half in domain III is most highly conserved containing two EF-hand domains in all organisms. EF-hand II domain in NlSPARC is stabilized by a disulfide bridge between cysteine residues 258 and 274 and an additional cysteine residue (286) located near the C-terminus which is found only in invertebrate SPARC proteins (Fig. 1). The name of the species, the corresponding accession numbers for SPARC, length and the pIs of Domain I are listed in Table S1.

To clarify the evolutionary relationship of NlSPARC, we used a neighbor-joining tree construction program Mega 7 based on distances of 18 SPARC sequences from 18 arthropod including Coleoptera, Lepidoptera, Diptera, Hemiptera, Dictyoptera, Hymenoptera and Psocodea. The dendrogram obtained places the NlSPARC with Hemiptera as a distinct cluster (Figure S1).

### Developmental and tissue-specific expression of *NlSPARC* in *N. lugens*

**Fig. 2:**
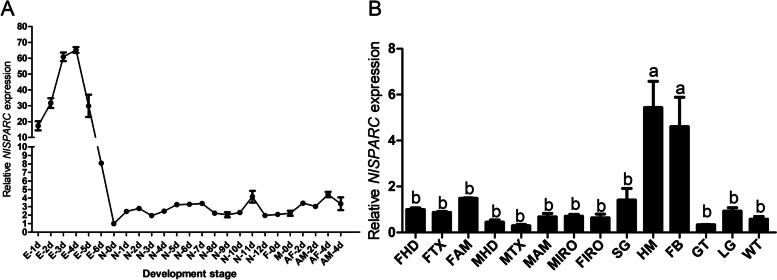
Relative expression levels of *NlSPARC* in *N. lugens*, as determined by real-time quantitative PCR (RT- qPCR). **A** Expression patterns of *NlSPARC* in different developmental stages including E-1d ~ E-6d (eggs from 1 to 6 days), N-1d ~ N-12d nymphs from 1 to 12 days, F-0d ~ AF-4d, newly emerged females, 2- and 4- days old adult females; M-0d ~ AM-4d, newly emerged males, 2- and 4- days old adult males. **B** Expression patterns of *NlSPARC* in various tissues including FHD, head from females; FTX, thorax from females; FAM, abdomen from females; MHD, head from males; MTX, thorax from males; MAM, abdomen from males; MIRO, internal reproductive organ of males; FIRO, internal reproductive organ of females; GT, guts; SG, salivary glands; HM, hemolymph; LG, legs; WT, wings and teguments. All data are presented as means ± SE with three biological replicates and relative expression levels of target genes were calculated by RT-qPCR with the 2^‒ΔΔCT^ method. Different lowercase letters above the bars indicate significant differences among different tissues by the Tukey’s multiple range test at *P* < 0.05

Egg and nymphal development normally lasts 6 ~ 7 days and 12 ~ 14 days, respectively. Nymphal development is comprised of five nymphal instars, from N1 to N5, spanning about 48 h for each. The RT-qPCR analysis revealed that *NlSPARC* was expressed at all developmental stages of *N. lugens*. Expression of *NlSPARC* was highest on average in 3- and 4-day-old eggs, then declined rapidly in 5- and 6-day-old eggs and reached to the lowest level in newly hatched nymphs (Fig. 2A). The results of the expression analysis of *NlSPARC* in various tissues showed that the highest expression was in fat body and hemolymph and there was no significant difference between females and males (Fig. 2B).

### Knock-down of *NlSPARC* resulted in nymph mortality

**Fig. 3:**
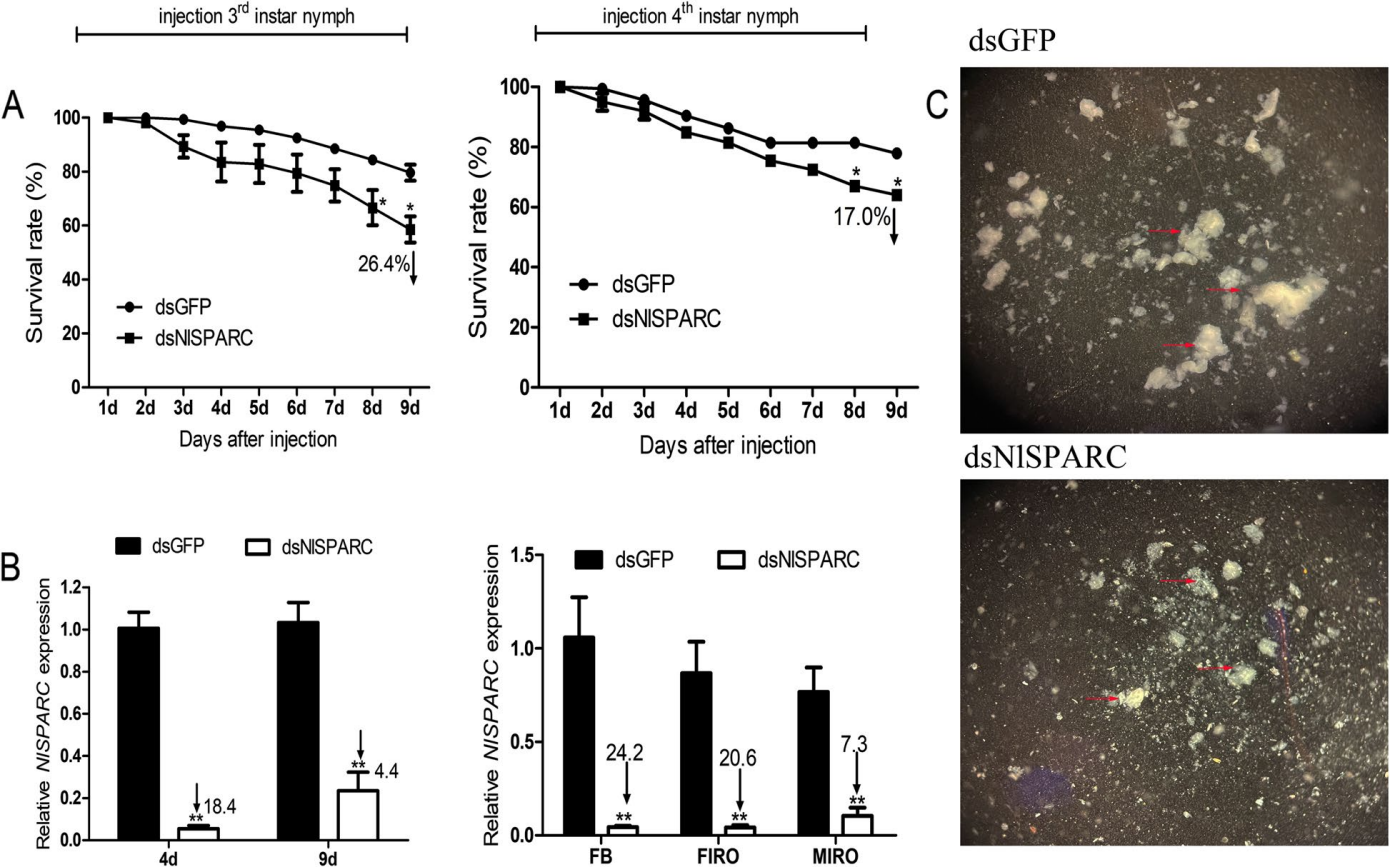
Effects of dsNlSPARC injection on nymph mortality (**A**), expression level of *NlSPARC* (**B**) and fat bodies (**C**). **A** injection the 3^rd^ instar nymphs and injection the 4^th^ instar nymphs; **B** reduction in mRNA expression level in whole body from injected the 3^rd^ instar nymphs and in fat bodies and internal reproductive organs from adults developed from injected the 4^th^ instar nymphs; **C** fat bodies dissect from adults developed from injected the 4^th^ instar nymphs. Fat bodies are highlighted with red arrow. The amount and volume of fat bodies were reduced in dsNlSPARC treatment compared to the control (dsGFP). All data are presented as means ± SE with four biological replications. *P < 0.05, ** *P* < 0.01

Based on the sequence alignment of *NlSPARC*, the regions (95–891 bp) of *NlSPARC* with great divergence from other insects were selected for the synthesis of dsRNA. Preliminary study conducted for dose-determination showed *NlSPARC* expression was downregulated by more than 70% and the nymph mortality within 24 h was less than 20% after injection of dsNlSPARC in nymphs with a concentration of 700 ng/μL (i.e., 70 ng dsRNA in each insect), which concentration was used for further study. In order to elucidate the function of *NlSPARC* in nymphal development, 3^rd^ instar and 4^th^ instar nymphs were injected with dsNlSPARC, respectively. There was a significant increase in nymph mortality at day 8 after injection. The mortality was 36.0% and 46.4% at day 9 after injection in 3^rd^ instar and 4^th^ instar nymphs respectively, whereas it was 20.4% and 22.1% in control with dsGFP (*P* = 0.0104, *P* = 0.0004, respectively) (Fig. 3A).

*NlSPARC* mRNA levels were measured in 3^rd^ instar nymphs after injection at day 4 and 9 and in tissues of adults developed from the injected 4^th^ instar nymphs. Compared with dsGFP injection, in the 3^rd^ instar nymphs, expression of *NlSPARC* significantly decreased by 18.4-fold at day 4 (*P* = 0.0003) and 4.4-fold at day 9 (*P* = 0.0035) after injection of dsNlSPARC (Fig. 3B). In the 4^th^ instar nymphs, expression of *NlSPARC* significantly decreased by 24.2-, 20.6- and 7.3-fold in fat body, female and male internal reproductive organs respectively (*P* = 0.0034, *P* = 0.0027 and *P* = 0.0088) (Fig. 3B). After insect anatomy evaluation, we found that the fat body in adults injected with dsNlSPARC was smaller and less abundant than those from adults injected with dsGFP (Fig. 3C).

### Effects of *NlSPARC* knock-down on reproduction and progeny

**Fig. 4:**
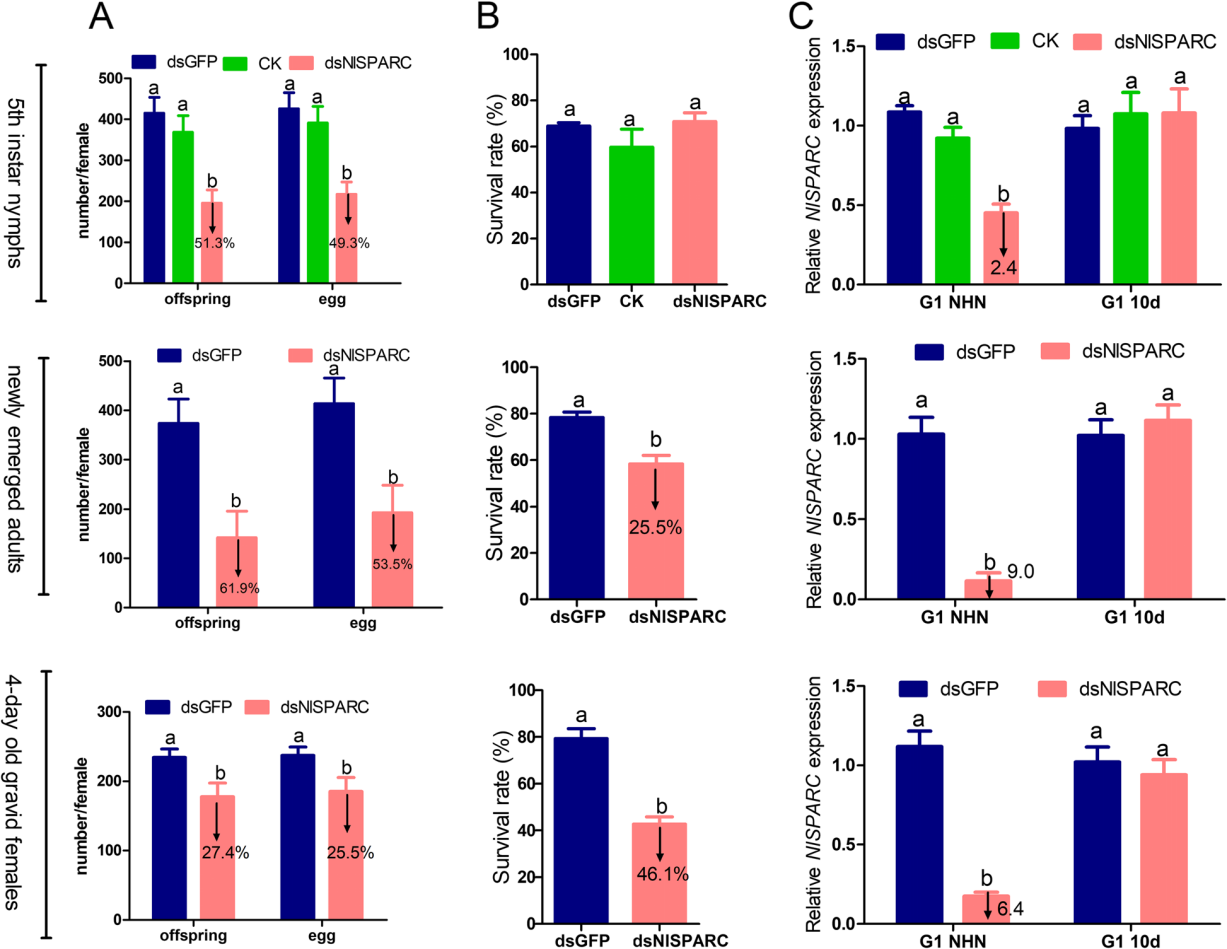
Effects of dsNlSPARC on fecundity (**A**), survival rate of progeny (**B**) and expression level of *NlSPARC* in *N. lugens* (**C**). The 5^th^ instar nymphs, newly emerged adults and 4-day old gravid females were injected with dsNlSPARC respectively. Individuals treated with dsGFP were used as a control. The number of offspring and egg was calculated from fifteen biological replicates (Mean ± SE). *NlSPARC* mRNAs levels of 30 new hatched nymphs and 20 10-day old mixed insects with 10 females and 10 males were analyzed by RT-qPCR with the 2^‒ΔΔCT^ method. Data is presented as mean ± SE with five biological replicates. G1NHN, newly hatched nymphs from F1 progeny; G1 10d, 10d-old nymphs from F1 progeny. Different lowercase letters and ** above the bars indicate significant differences at *P* < 0.05 and *P* < 0.01 respectively among different treatments by the Tukey’s multiple range tests.

In order to elucidate the function of *NlSPARC* in *N.lugens* reproduction, the 5^th^ instar nymph, newly emerged adults and gravid females were injected with dsNlSPARC, respectively. As no difference between injection of dsGFP and no injection control in 5^th^ instar nymphs was observed, the no injection control was omitted in other stages. The injection of dsNlSPARC did not significantly affect adult longevity and ovariole maturation. The number of offspring after injection of dsNlSPARC in the 5^th^ instar nymph, newly emerged adults and gravid females was significantly reduced by 51.3%, 59.1% and 52.5%, respectively (*P* = 0.0001, *P* = 0.0174 and *P* = 0.0300). The number of eggs after injection of dsNlSPARC in the 5^th^ instar nymph, newly emerged adults and gravid females was significantly reduced by 49.3%, 59.2% and 46.0%, respectively (*P* = 0.0001, *P* = 0.0252 and *P* = 0.0467), compared to dsGFP injection (Fig. 4A). No difference in hatch rate between the treatment and control was found (data not shown).

There was no significant difference in 10-day survival rate of F1 progeny between injection of dsNlSPARC and dsGFP in 5^th^ nymphs. The 10-day survival rate of F1 progeny of newly emerged adults injected with dsNlSPARC was 58.3%, and that of F1 progeny of gravid females injected with dsNlSPARC was 42.6%. Compared with dsGFP control, it was significantly reduced by 25.5% and 46.1%, respectively (*P* = 0.1956 and *P* = 0.0003) (Fig. 4B). Most nymphs die at the 1-2^nd^ instar stage. No obvious morphological abnormalities were observed in injected individuals and their progeny.

*NlSPARC* mRNA levels were measured in F1 progeny with RNA extracted from 30 newly hatched nymphs and 20 mixed insects of 10 females and 10 males. Compared to dsGFP-injected control, the expression of *NlSPARC* decreased significantly in newly hatched nymphs by 2.4, 9.0 and 6.4 times after injection of the 5^th^ instar nymphs, newly emerged adults and gravid females, respectively (*P* < 0.0001),whereas 10 days later no significant difference between treatment and control was found (Fig. 4C).

mRNA levels of *NlSPARC*, *NlVg* (Vitellogenin), *NlVgR* (Vitellogenin receptors) and *NlFOXO* were also measured in newly emerged females developed from the injected 5^th^ instar nymphs. Compared to dsGFP-injected control, the expression of *NlSPARC* decreased by 7.0 times and the expression of *NlVg*, *NlVgR* and *NlFoxO* showed no significant difference (Figure S2).

**Fig. 5:**
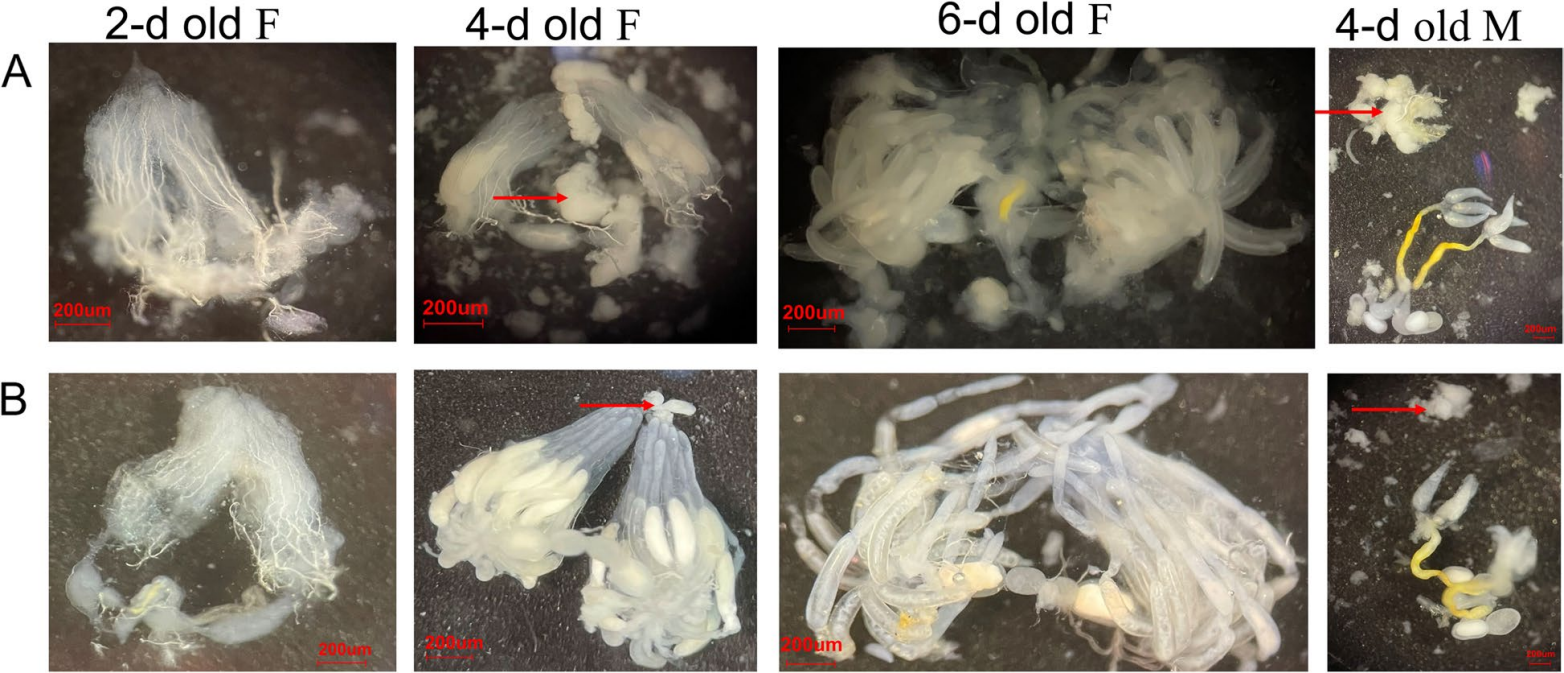
Effects of dsNlSPARC on the internal reproductive organs. **A** 2-,4- 6- day old females (F) and 4 day old males (M) with dsGFP treated *N.lugens*; **B** 2-,4- 6- day old females (F) and 4 days old males (M) with dsNlSPARC treated *N.lugens*. Fat bodies are highlighted with red arrow. No obvious difference in development of internal reproductive organ, ovarian tubules and egg granules were found between the treatment injected with dsNlSPARC and the control injected with dsGFP. The ovarian tubules were easily dispersed and the amount and volume of fat body were reduced in dsNlSPARC treatment compared to the control (dsGFP)

As the number of offspring decreased after injection of dsNlSPARC, we then dissected the adults and found that the ovarian tubules were easily dispersed and the amount and volume of fat body reduced compared to the control (dsGFP) (Fig. 5). There was no obvious difference in development of internal reproductive organ, ovarian tubules and egg granules between dsNlSPARC and dsGFP treatment (Fig. 5).

## Discussion

SPARC is a secreted calcium-binding and collagen-binding glycoprotein that has functions in tissues remodelling during development [16]. Genome analysis has revealed a single copy of the *Drosophila SPARC* gene [13]. *NlSPARC* is encoded by a single gene and located in chromosome 2 in *N.lugens*. Comparative analysis of primary sequences of SPARC proteins from diverse species revealed functional conservation, including the high-affinity Ca^2+^ binding sites in domain III. As reported in *Drosophila* [13, 17], temporal and spatial expression data in *N.lugens* indicated that maximum levels of SPARC expression occur during embryonic development stages and in fat body and hemocytes.

Injection of dsNlSPARC in the 3^rd^ or 4^th^ instar nymphs caused a significant decrease in *NlSPARC* transcripts at 4 and 9 days after injection, especially in fat bodies and female internal reproductive organs. This result suggests that the genes expressed in fat body and internal reproductive organs can be down-regulated by RNAi in *N.lugens*. A notable result was that a significant reduction in *NlSPARC* expression was observed in nearly all newly hatched nymphs of the progeny after injection of dsNlSPARC in the 5^th^ instar nymphs and adults, and 10 days later, the expression level returned to the same level as the control. This indicated that *NlSPARC* RNAi is still in effect in the next generation in *N.lugens*. This phenomenon was called parental RNAi (pRNAi), which is identified by determining whether dsRNA introduced into the body cavity resulted in gene inactivation in offspring embryos [20, 21]. The phenomenon of pRNAi, especially the genes involved in embryonic development has been confirmed in *Tribolium castaneum* [22], *Acyrthosiphon pisum* [23], *Diabrotica virgifera virgifera* and *Euschistus heros* [24]. Our result showed the pRNAi of *SPARC* also exists in *N.lugens*.

Down-regulation of the *NlSPARC* with 18.4-folds in 3^rd^ instar nymphs resulted in the corrected mortality of 26.4%, with 6.4- and 9.0- folds in new hatched nymphs of F1 progeny resulted in the corrected mortality of 46.1% and 25.5%. With a 2.4-folds down-regulation of *NlSPARC* in new hatched nymphs of F1 progeny did not result signifiant mortality. The down-regulation fold of *NlSPARC* expression was lower in newly hatched nymphs than in 3^rd^ instar nymphs, whereas the effect on mortality was more significant.In *Drosophila*, loss of SPARC alone leads to defective fat body basal laminae assembly, embryonic lethality and larval lethality of the 2^nd^ instar [14, 16, 25]. In *C. elegans*, a reduction in the amount of SPARC protein also resulted in embryonic or larval lethality in a substantial proportion of progeny, and no significant morphological abnormalities were observed [11].

In nematodes, developmental defects were observed during embryogenesis following SPARC deletion, while there appeared to be no changes in adult gonad or oocyte morphology, suggesting that the fertilization process was disturbed [11]. In *D.melanogaster*, there was an increase of its expression during previtellogenic stages, which indicated that SPARC play a role in oocyte development and vitellogenesis [13]. In *Blattella germanica*, SPARC is necessary to maintain the cytoskeleton of the follicular cells and depletion of *SPARC* disables females for oviposition [19]. Therefore, we selected three different developmental stages including the 5^th^ instar nymphs, newly emerged adults and gravid females for RNAi to study the function of this gene in *N.lugens* reproduction. Knock-down of the *NlSPARC* in these stages resulted in a reduction of offspring and egg number, whereas the egg hatchability, early embryo development was not affected. The expression of genes *NlVg*, *NlVgR* and *NlFoxO,* which related closely to oocyte development and vitellogenesis, was not changed [26, 27]. This result implied that the fertilization process and vitellogenesis were not affected after knock-down the *NlSPARC*. Microanatomy showed that down-regulation of *NlSPARC* resulted in the decrease of the fat body, which is crucial for development and acts as the primary source of energy. Together with hemocytes, fat body is the major sources of basal laminae components during larval development [28]. This result showed that oviposition events and processes are affected after knock-down of *NlSPARC* in *N.lugens* and this effect may be mainly achieved by regulating the fat body. More detailed experiments and further analyses of SPARC function are needed to reveal its precise role in the *N.lugens*.

Moderate control of population densities can help delay the rate of resistance evolution and is therefore considered a greener, gentler strategy for sustainable control. RNAi has been widely applied as a genetics tool for gene function analysis through the sequence-specific suppression of target gene. It is also regarded as a potential approach for insect pest management as it avoids the rapid formation of resistant populations and excessive use of chemical pesticides [29, 30]. Recently report showed that the sap-sucking insect pest can be effectively controlled by plastid-mediated RNAi including *N.lugens* [31]. RNAi of *NlSPARC* showed mortality, especially in early nymphal stages and low fecundity. Although the decline in survival and the number of offspring was not drastic after RNAi of *NlSPARC* in *N.lugens*, when it fed on transplastomic rice expressing dsNlSPARC, the suppression of *NlSPARC* expression persisted throughout the developmental period and across successive generations, the population of *N. lugens* will be controlled within a certain range. Compared with the gene with high mortality after down-regulation, *NlSPARC* is more suitable as a candidate target gene for green control of *N.lugens* through RNAi, which meets the standards of green control by reducing initial population sizes, decreasing population growth rates of pest and controlling pests within the allowable economic loss density without affecting the biodiversity in rice fields [32]. Considering the high similarity of domains II and III of SPARC proteins in insects and thus possibly causing off-target effects in RNAi applications, designing dsRNA against the sequence of domain I can improve the specific selectivity for the control of the target pest *N. lugens*.

## Materials and methods

### Insects and tissue sampling

*N. lugens* were reared on rice variety Taichung Native 1 at 26 ºC ± 2 ºC under a 16 h light/8 h dark cycle. Six developmental stages: Eggs (200) within 1 ~ 6 days, and the individuals (50) from the first day of the 1^st^ instar to the twelfth day of the 5^th^ instar nymphs, newly emerged females (10) and male adults (10) as well as 4- day old females and males (10) were randomly selected. Adult females and males at 2 days after eclosion were immobilized by placing them in a freezer for 15 min, tissues including guts, salivary glands, fat bodies, hemolymph, legs, wings and teguments from 50 to 100 mixed sex adults were dissected and internal reproductive organ from males (50) and females (50). Heads (20), abdomens (20) and thoraces (20) from males and females respectively were dissected with tweezers. The number of insects in each sample was given in parentheses above. All samples were collected in triplicate. The samples were frozen in liquid nitrogen and stored at -80 ºC prior to RNA extraction.

### RNA extraction, cDNA preparation and RT-qPCR analysis

Total RNA extraction, cDNA synthesis, T-A clone and RT-qPCR analysis were conducted according to our previous study [33]. Total RNA was isolated from the whole bodies or tissues of *N.lugens* using RNeasy Mini Kit (Qiagen, Valencia, CA) following the manufacturer’s recommendations. Total RNA (0.5-1 µg) was used to synthesize first strand cDNA with ReverTra Ace qPCR RT Kit (Toyobo Co., Ltd., Japan). RT-qPCRwas performed using SYBR green mix and a thermal cycler CFX-96 PCR detection system (Bio-Rad, Philadelphia, PA, USA). The primers used for clone are NlSPARC-F: GACATTCCTCAGTCCACGAGT and NlSPARC-R: ATACGGTTACTGGGTTATGAACA. *NlSPARC* specific primers for RT-qPCR expression are qNlSPARC-F: ACCTCTCCACCTCCCGATTT and qNlSPARC-R: TGCAAACACACTTTGCCTTCT. Primers were synthesized by Shangya Biotechnology Co. RT-qPCR analysis was performed with at least three biological replicates, and three technical replications for each. *Nlactin* and *Nl18S rDNA* were used as the reference gene to normalize gene transcript levels [34]. Relative quantification of the transcripts was calculated by the 2^‒ΔΔCT^ method [35].

### Phylogenetic studies

The open reading frame (ORF) for *NlSPARC* was predicted using DNASTAR software (DNASTAR Inc., Madison, USA) and the corresponding amino acid sequence of *NlSPARC* was analyzed using SignalP 3.0 (http://www.cbs.dtu.dk/services/ SignalP-3.0). The sequence domains were identified using the Conserved Domain Search Service from NCBI. Sequences used in the phylogenetic analysis were obtained by Blast from GenBank, using the SPARC protein sequences from *N.lugens*, *B. germanica* and *D.melanogaster* as query. A phylogenetic tree was constructed by MEGA version 7.0 (http://megasoftware.net/) using the neighbor-joining method. A Poisson-corrected distance was used and the data was bootstrapped for 1000 replicates.

### dsRNA synthesis and RNAi experiments

To knock-down *NlSPARC* for assessing the specificity of the phenotype, the dsRNA was synthesized in vitro using the MEGA script high-yield transcription kit (Applied Biosystems Inc., USA). And then quantified using a NANODROP™ 1000 spectrophotometer (Thermo Scientific, Franklin, MA) at 260 nm and analyzed by gel electrophoresis to determine purity. The primers used for dsNlSPARC were dsNlSPARC-F: TAATACGACTCACTATAGGG GAATAGACAGGCCGAGCAGTG and dsNlSPARC-R: TAATACGACTCACTATAGGG TGCAATGTTCCAGAGCCATGA. A dsGFP that targets the green fluorescent protein (GFP) gene (AB608314) was also produced and used as control.

The dsNlSPARC or dsGFP was injected into the 3^rd^ instar, 4^th^ instar, 5^th^ instar nymphs, newly emerged adult insects and gravid 4-day old females respectively according to the method described by Zhang et al.[36]. Gravid 4-day old females were collected as follow: virgin males and females (24 h old) were allowed to mate for 4 days. On day five, males were removed and the remaining females were used for injection. Each nymph was injected with 0.1μL dsRNA solution into the thorax. Each adult insect was injected into the thorax with 0.2 μL dsRNA solution. The efficiency and specificity of RNAi were examined by RT-qPCR at day 4 or 9 after injection of the 3^rd^ instar nymphs, different tissues of adults developed from injected the 4^th^ instar nymphs or newly hatched nymphs from progeny of each paired parent.

For investigation of the survival, at least 30 individual nymphs were injected and reared on 30- to 35-day-old rice of Taichung Native1 in one cage at 28℃, 85% RH, and 16:8 (L: D) h darkness with three separated parallel replicates. The number of surviving nymphs was counted daily until 9 days after injection. The survival rate was calculated as the number of live insects divided by the starting number of insects. For investigation of the fecundity, each newly emerged female and male after injection were reared in one cage with 60-day-old rice of Taichung Native1 and allowed to oviposit until parents die. At least 15 pairs were successfully paired. Each gravid 4-day old female after injection was reared in one cage and allowed to oviposit for seven days. At the 7^th^ day the females were collected to dissect under the stereomicroscope. Then the number of nymphs hatching from each female was counted on rice stems for 10–12 consecutive days until no further hatching was observed. Finally, rice stems were dissected and examined with a stereomicroscope, and the number of eggs was counted. At least fifteen biological replicates were used for statistical analysis.

### Data analysis

The differences between control and RNAi treatments were analyzed using one-way analysis of variance followed by the Tukey’s multiple range tests for multiple comparisons using DPS software [37].

## Supplementary Information

**Additional file 1: Table S1.** Information of protein sequences used in alignment.

**Additional file 2: Figure. S1.** Unrooted phylogenetic tree of NlSPARC from *N.lugens* and representative insect species. An unrooted phylogenetic tree was constructed by the neighbour-joining tree construction program Mega 7. Evolutionary distances were computed using Poisson correction method. Branch support values (1000 bootstraps) for nodes are indicated only support values > 50% are shown. NlSPARC is marked with filled triangle. All protein sequences (accession numbers, length and pI) obtained from GenBank was listed in Table S1.

**Additional file 3: Figure. S2.** Relative expression of *NlSPARC*,*NlVg*,*NlVgR*, and *NlFoxO *in newly emerged adults developed from injected 5^th^ instar nymphs. mRNA levels of *NlSPARC*, *NlVg,* *NlVgR*, and *NlFoxO *from 5 newly emerged females were analyzed by RT-qPCRwith the 2^‒ΔΔCT^ method from three biological replicates (Mean ± SE). ** above the bars indicate significant differences at P < 0.01 among different treatments by the Tukey’s multiple range tests.

## Data Availability

The datasets generated and/or analysed during the current study are available in the [GenBank] repository, [GenBank https://www.ncbi.nlm.nih.gov/gene under accession number MZ983402].
