# Supplementary material for: SPARC plays an important role in the oviposition and nymphal development in Nilaparvata lugens Stål
Source: BMC Genomics. 2022 Oct 3;23:682. doi: 10.1186/s12864-022-08903-z (PMC9531499; doi:10.1186/s12864-022-08903-z)
Supplement: Supplementary file 1 — Additional file 1: Table S1. Information of protein sequences used in alignment. [file 12864_2022_8903_MOESM1_ESM.docx]

Table S1. Information of protein sequences used in alignment.

| Species name | Accession NO. | Length（aa） | PI of domain I |
| --- | --- | --- | --- |
| *Nilaparvata lugens* | MZ983402 | 297 | 6.3 |
| *Diabrotica virgifera virgifera* | XP_028135584.1 | 290 | 5.8 |
| *Tribolium castaneum* | XP_975465.1 | 293 | 6.4 |
| *Leptinotarsa decemlineata* | XP_023029545.1 | 321 | 4.7 |
| *Blattella germanica* | CZQ50751.1 | 320 | 4.6 |
| *Zootermopsis nevadensis* | KDR16367.1 | 321 | 4.5 |
| *Culex quinquefasciatus* | XP_001847806.1 | 321 | 3.7 |
| *Aedes aegypti* | XP_001663842.1 | 322 | 3.8 |
| *Drosophila melanogaster* | NP_651509.2 | 304 | 4.2 |
| *Laodelphax striatellus* | RZF43002 | 297 | 6.2 |
| *Apis mellifera* | XP_623079.1 | 304 | 5.3 |
| *Megachile rotundata* | XP_003701092.1 | 307 | 4.8 |
| *Orussus abietinus* | XP_012283372.1 | 301 | 4.5 |
| *Nasonia vitripennis* | XP_001600190.1 | 297 | 5.0 |
| *Bombyx mori* | NP_001040421.1 | 317 | 4.8 |
| *Manduca sexta* | XP_030023102.1 | 322 | 5.2 |
| *Pediculus humanus corporis* | XP_002428886.1 | 272 | 3.9 |
| *Acyrthosiphon pisum* | NP_001155422.1 | 300 | 9.7 |

Figure. S1. Unrooted phylogenetic tree of NlSPARC from *N.lugens* and representative insect species. An unrooted phylogenetic tree was constructed by the neighbour-joining tree construction program Mega 7. Evolutionary distances were computed using Poisson correction method. Branch support values (1000 bootstraps) for nodes are indicated only support values > 50% are shown. NlSPARC is marked with filled triangle. All protein sequences (accession numbers, length and pI) obtained from GenBank was listed in Table S1.

Figure.S2. Relative expression of *NlSPARC*,*NlVg*,*NlVgR*, and *NlFoxO* in newly emerged adults developed from injected 5^th^ instar nymphs. mRNA levels of *NlSPARC*, *NlVg,* *NlVgR*, and *NlFoxO* from 5 newly emerged females were analyzed by RT-qPCRwith the 2^‒ΔΔCT^ method from three biological replicates (Mean ± SE). ** above the bars indicate significant differences at P < 0.01 among different treatments by the Tukey’s multiple range tests.
