# Supplementary figures and images for: SPARC plays an important role in the oviposition and nymphal development in Nilaparvata lugens Stål
Source: BMC Genomics. 2022 Oct 3;23:682. doi: 10.1186/s12864-022-08903-z (PMC9531499; doi:10.1186/s12864-022-08903-z)

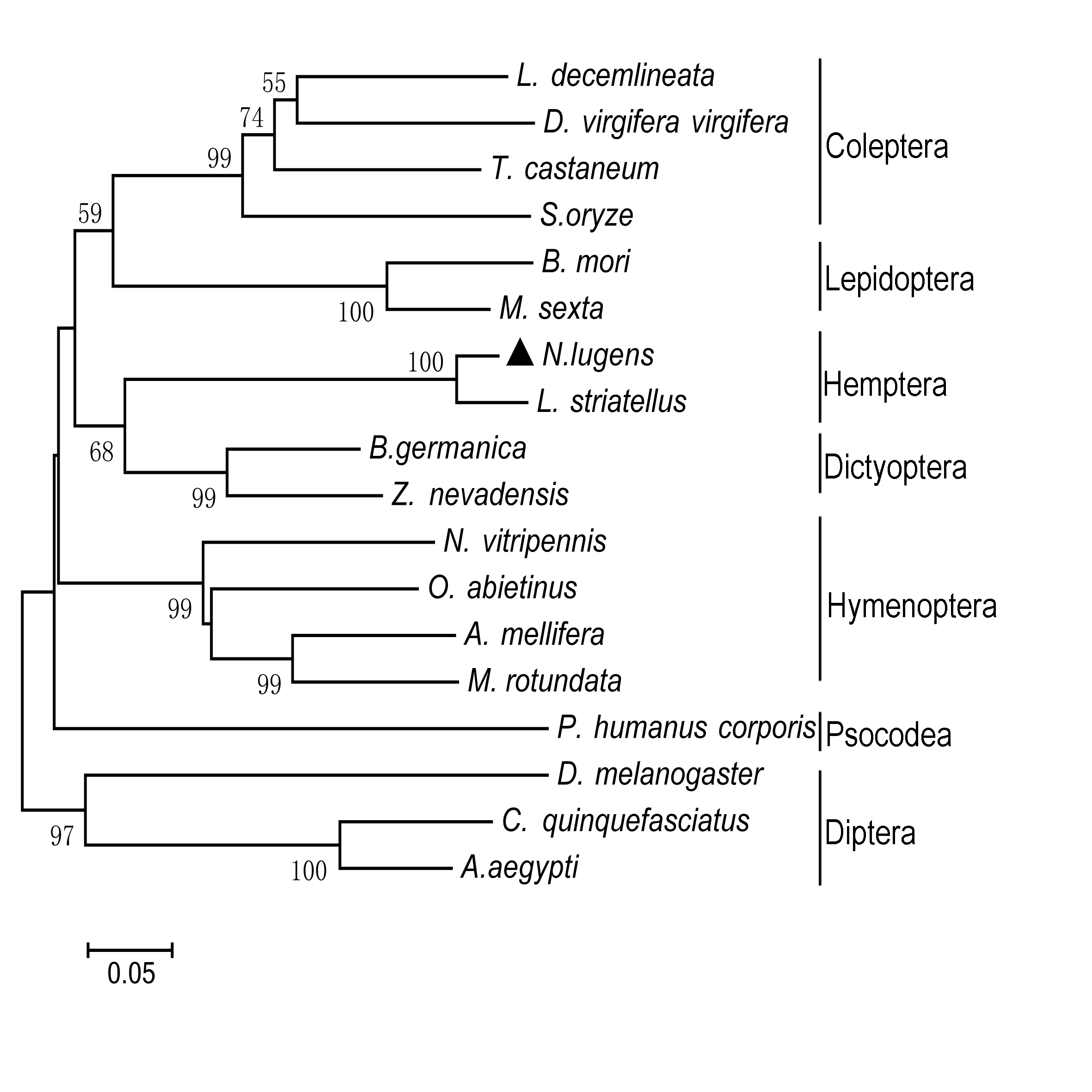

Supplement: Supplementary file 2 — Additional file 2: Figure. S1. Unrooted phylogenetic tree of NlSPARC from N.lugens and representative insect species. An unrooted phylogenetic tree was constructed by the neighbour-joining tree construction program Mega 7. Evolutionary distances were computed using Poisson correction method. Branch support values (1000 bootstraps) for nodes are indicated only support values > 50% are shown. NlSPARC is marked with filled triangle. All protein sequences (accession numbers, length and pI) obtained from GenBank was listed in Table S1. [file 12864_2022_8903_MOESM2_ESM.tif]
